# A Simple LC-MS/MS Method for the Quantification of PDA-66 in Human Plasma

**DOI:** 10.3390/molecules27030974

**Published:** 2022-02-01

**Authors:** Rico Schwarz, Elisabeth R. D. Seiler, Sina Sender, Anahit Pews-Davtyan, Hugo Murua Escobar, Dietmar Zechner, Matthias Beller, Christian Junghanß, Burkhard Hinz

**Affiliations:** 1 Institute of Pharmacology and Toxicology, Rostock University Medical Centre, 18057 Rostock, Germany; rico.schwarz@med.uni-rostock.de (R.S.); e-seiler@eagle.sophia.ac.jp (E.R.D.S.); 2 Clinic for Hematology, Oncology and Palliative Care, Rostock University Medical Centre, 18057 Rostock, Germany; sina.sender@med.uni-rostock.de (S.S.); hugo.murua.escobar@med.uni-rostock.de (H.M.E.); christian.junghanss@med.uni-rostock.de (C.J.); 3 Leibniz Institute for Catalysis e. V. at the University of Rostock, 18059 Rostock, Germany; anahit.pews-davtyan@davtyanlaw.com (A.P.-D.); matthias.beller@catalysis.de (M.B.); 4 Rudolf Zenker Institute for Experimental Surgery, Rostock University Medical Centre, 18057 Rostock, Germany; dietmar.zechner@med.uni-rostock.de

**Keywords:** PDA-66, human plasma, LC-MS/MS, validation, liquid–liquid extraction

## Abstract

The treatment of cancer is one of the most important pharmacotherapeutic challenges. To this end, chemotherapy has for some time been complemented by targeted therapies against specific structures. PDA-66, a structural analogue of the inhibitor of serine–threonine kinase glycogen synthase kinase 3β SB216763, has shown preclinical antitumour effects in various cell lines, with the key pathways of its anticancer activity being cell cycle modulation, DNA replication and p53 signalling. For the monitoring of anticancer drug treatment in the context of therapeutic drug monitoring, the determination of plasma concentrations is essential, for which an LC-MS/MS method is particularly suitable. In the present study, a sensitive LC-MS/MS method for the quantification of the potential anticancer drug PDA-66 in human plasma with a lower limit of quantification of 2.5 nM is presented. The method was successfully validated and tested for the determination of PDA-66 in mouse plasma and sera.

## 1. Introduction

**Figure 1:**
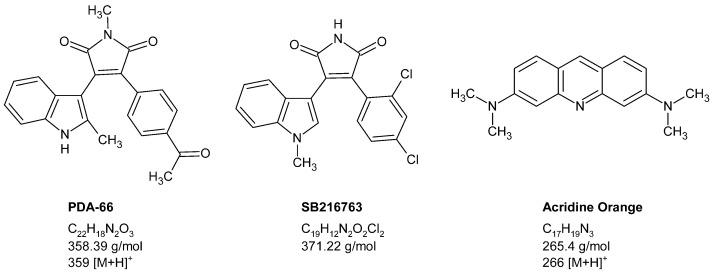
Chemical structures, molecular formulae and molecular weights of PDA-66, SB216763 and the internal standard acridine orange. In the case of PDA-66 and acridine orange, the precursor ions are also indicated.

The treatment of different types of cancer has been the focus of scientific interest for decades. Today, conventional chemotherapy is complemented by new therapeutic approaches against specific structures and signalling pathways. One example of potentially new targets for cancer therapy is the serine–threonine kinase glycogen synthase 3β (GSK3β), for which the maleimide derivative SB216763 has been described as an inhibitor [1]. Based on the antitumour activity of SB216763, a structural analogue, PDA-66 (Figure 1), was developed, which, compared to SB216763, has an unprotected 2-methylindole moiety, a methylated maleimide group, and a 4-acetyl group instead of the 2,4-dichloro substituent [2]. Initial preclinical studies with PDA-66 showed antiproliferative and proapoptotic effects on human progenitor and cancer cells [3]. Later, these effects were confirmed in acute lymphoblastic leukaemia [4] and canine lymphoma cells [5], the latter being a model very similar to human high-grade non-Hodgkin’s lymphoma. Despite its chemical similarity to SB216763, PDA-66 has not demonstrated significant inhibition of GSK3β [4]. On the other hand, whole transcriptome sequencing analyses have clearly shown that the key pathways of PDA-66’s antitumour activity are cell cycle modulation, DNA replication and p53 signalling [5]. The exact mechanisms of antitumour activity as well as the pharmacokinetics of PDA-66 are currently under further investigation. In particular, kinetic studies require the establishment of a reliable method to quantify the substance from plasma or serum.

Liquid chromatography–tandem mass spectrometry (LC-MS/MS) is ideal for the identification and quantification of substances from a wide variety of matrices. Soft ionisation techniques, such as electrospray ionisation, enable molecules to be charged whilst ensuring minimal fragmentation of the parent compound prior to measurement in the first quadrupole. Afterwards, collision-induced dissociation can be used to fragment the resulting ions, and a third quadrupole provides a sensitive determination of the generated fragments. In the present study, therefore, an LC-MS/MS method for the quantitative determination of PDA-66 in human plasma was developed and validated according to the U.S. Food and Drug Administration (FDA) guidelines [6].

## 2. Results and Discussion

### 2.1. Calibration Curve

**Figure 2:**
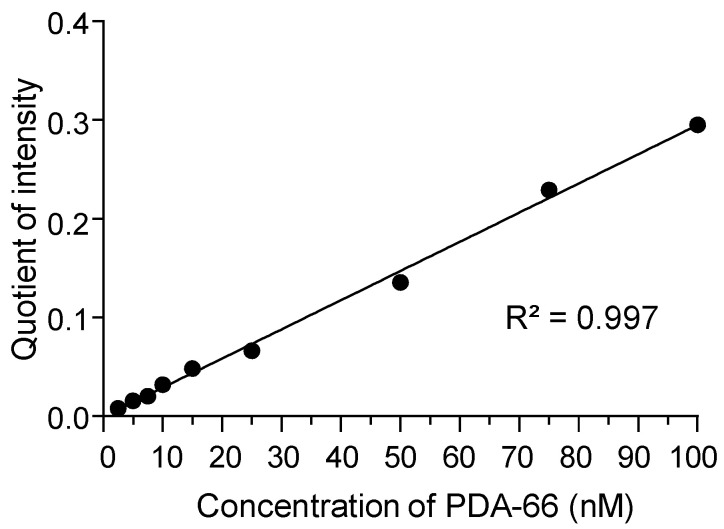
Sample calibration function, in which the quotient of the area of the quantifiers *m/z* 317.1 (PDA-66) and *m/z* 250.1 (internal standard) was formed at the indicated concentrations.

The concentrations of PDA-66 in the calibration curve in human plasma ranged from 2.5 nM to 100 nM. The peak area ratios of the MS/MS fragments of the internal standard and the PDA-66 quantifiers were determined and linear regression was performed. The coefficient of determination was at least 0.99 or better in all validation runs (Figure 2). The low concentration range was chosen to minimise the use of human or mouse plasma for the analysis of PDA-66 in perspective measurements.

### 2.2. Selectivity, Specificity and Carryover

**Figure 3:**
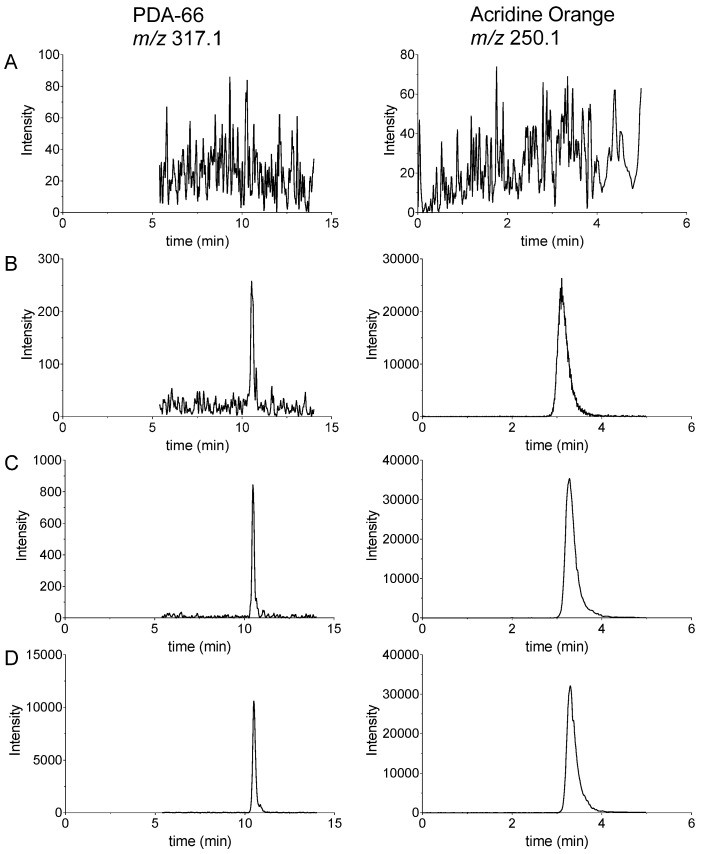
Chromatograms from LC-MS/MS analysis of (**A**) a blank sample, (**B**) a sample containing 2.5 nM PDA-66 (LLOQ), (**C**) a 7.5 nM PDA-66 standard sample and (**D**) a 100 nM PDA-66 standard sample. Shown are exemplary chromatograms of the quantification ions for PDA-66 and acridine orange (internal standard). Not shown are the qualifier ions for PDA-66 (*m/z* 230.2) and the internal standard (*m/z* 234.1).

In the measurement of a total of six different human plasmas carried out to prove the selectivity, no MS/MS signals could be detected in the range of the retention times of PDA-66 and its internal standard acridine orange (Figure 3A). The selectivity of the method could thus be confirmed.

To investigate the specificity of the method, PDA-66 was added to a 10% S9 mouse and human liver solution with a final PDA-66 concentration of 10 µM. An S9 liver lysate solution contains the majority of phase I and phase II enzymes that metabolise drugs, and is therefore ideal for general in vitro drug metabolism studies [7]. After incubation at 37 °C for 3 h, the solution was extracted and analysed by LC-MS. 

A full scan in the mass range from *m/z* 250 to *m/z* 750 was chosen to analyse any high-abundance metabolites that might be co-extracted with PDA-66. Thereby, no clearly abundant signals could be obtained. Strikingly, little of the parent compound was seen after 3 h of incubation, suggesting enhanced metabolism by liver enzymes. Since our analysis was performed in full scan mode, it is possible that unstable metabolites such as glucuronides or sulphates resulted in the same *m/z* values, due to possible fragmentation in the ion source (for a detailed review see [8]). Especially in short LC-MS runs, an unstable metabolite may lead to incorrect results. In order to largely exclude this possibility, a long gradient was used in the method validated here. Furthermore, the very specific MRM mode [8] was chosen for quantification. Therefore, it is highly unlikely that an unstable metabolite would co-elute with PDA-66 and form the identical precursor ion and fragment ions. In addition, it should be noted that the metabolising enzymes of the liver most likely provide PDA-66 with hydrophilic groups, such as hydroxide functions, which would lead to a less efficient lipophilic extraction. Overall, the potential occurrence of stable or unstable metabolites could not be characterised here, but should be investigated in future studies.

According to Figure 3A, the carryover of PDA-66 and the internal standard from one LC-MS/MS run to the next was not observed throughout the calibration function. Furthermore, Figure 3 shows the chromatograms obtained from the lower limit of quantification (LLOQ) analysis (panel B), a 7.5 nM standard (panel C) and a 100 nM standard (panel D). The column efficiency (N) from the chromatograms shown was 21886.2 ± 4299.3 for PDA-66 and 1176.0 ± 122.7 for the internal standard (mean ± SD with *n* = 3 in each case). The column efficiency N was determined as follows: 5.54 × (t_R_/w_0.5_)^2^ where t_R_ is the *retention time* and w_0.5_ is the *peak width* at *half height*. A slight retention time shift, most likely caused by the lipid content of the plasmas, did not affect the determination of the two analytes.

### 2.3. Sensitivity, Accuracy and Precision

A concentration of 2.5 nM was determined as the LLOQ. This corresponds to 2 pmol or 0.72 ng of absolute substance at an injection volume of 80 µL. The signal-to-noise ratio (S/N) of the LLOQ was at least 5 in each validation run, which corresponds to the FDA guideline [6].

The inter- and intra-day accuracy (expressed as relative error, RE) and precision (determined as coefficient of variation, CV) for the LLOQ, 7.5 nM (low concentration), 50 nM (medium concentration) and 100 nM (high concentration) are shown in Table 1. The FDA requirements that the relative deviations in each validation run should not exceed ±15%, except for the LLOQ at ±20% [6], were always met. The CV must also not deviate by more than 15% according to FDA guidelines, with the exception of LLOQ at 20% [6]. This criterion was also met for all three concentration levels for both the intra-day and inter-day specifications. As shown in Table 1, the RE and CV values of the measured concentrations were below 10%, with the only exception being the 100 nM measurement on day 1, which was slightly above 10% at 10.32%, but was still within the required 15% of the guideline value. 

### 2.4. Matrix Effect and Recovery

A number of studies have shown that plasma components, such as endogenous substances, can negatively influence the ionisation of other components in electrospray ionisation (ESI) (for review see [9]). Therefore, the matrix effect plays an important role in the measurement of analytes. The matrix effect was analysed at three PDA-66 concentrations using five different samples (Table 2). A separate investigation was carried out to assess the matrix effect of the internal standard, acridine orange. To this end, the absolute values of the areas of extracted matrix samples, which were subsequently spiked with PDA-66, were compared with the areas of non-extracted PDA-66 standards. The result was a suppression of analyte ionisation. For PDA-66, it ranged from 31.0% ± 9.1% (7.5 nM) to 28.3% ± 14.7% (100 nM) to 28.0% ± 9.3% (50 nM), with a mean ± SD of *n* = 5 given here. Thus, the matrix effect reduces the final measurable concentration of PDA-66 in plasma by about 30%. The matrix effect for the internal standard acridine orange was 21.4% ± 5.8% (*n* = 5). However, this negative effect on the ionisation of the internal standard by the matrix had no influence on the measurements in general.

For recovery, the areas of samples spiked with PDA-66 and subsequently extracted were compared with those of extracted matrix samples that were spiked with the analyte only after extraction (Table 2). In both preparations, the internal standard was added after the extraction. For 7.5 nM PDA-66, the mean recovery was 102.0% ± 6.8% (mean ± SD) with a CV of 6.62% (*n* = 5). For the higher concentrations of 50 nM and 100 nM, the mean recoveries were 79.0% ± 7.4% and 85.0% ± 6.7% (mean ± SD, *n* = 10 each), respectively. Thus, recoveries at higher concentrations were reduced by a maximum of 21%. The precision for these two concentrations was 9.31% and 7.88%, respectively. Thus, with liquid–liquid extraction, PDA-66 can be largely extracted and recovered in different plasmas over the entire concentration range. Furthermore, the influence of five different matrices on the recovery of the internal standard acridine orange was investigated, which was necessary because no isotopically labelled PDA-66 is yet available. Here, the mean recovery was 65.89% ± 5.3% (mean ± SD) with a CV of 7.98% (*n* = 5). The internal standard was found in the analysed matrices without strong fluctuations, and is thus suitable for the determination of PDA-66.

### 2.5. Stability

**Figure 4:**
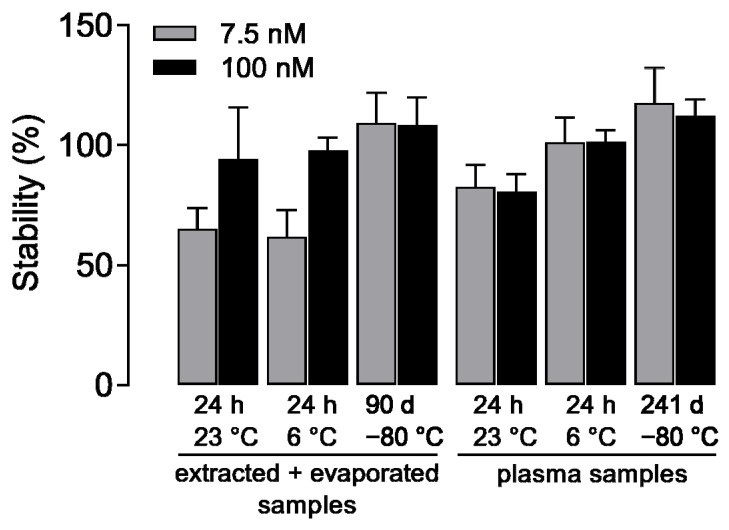
Stability of PDA-66 as a function of time and temperature of storage, relative to controls freshly extracted and co-determined on the day of measurement (set to 100%). The ratio of the *m/z* values of the quantifiers *m/z* 317.1 (PDA-66) and *m/z* 250.1 (internal standard) was determined. Shown are the mean values ± SD of *n* = 8 (90 days, −80 °C extracted and evaporated samples) and *n* = 4 (remaining analysed samples) independent LC-MS/MS runs.

Low and high concentrations of PDA-66 were exposed to different temperatures and processes to determine stability. Samples were stored for 90 days (extracted and evaporated samples) or 241 days (plasma samples) at −80 °C for the determination of long-term stability and for 24 h at either 23 °C (room temperature) or 6 °C for the determination of short-term stability. For these investigations, PDA-66 was stored as both a plasma sample and after extraction. At least three freshly extracted samples were always used for comparison. For comparison with the extracted storage samples, the internal standard was added to the reference samples after extraction. Figure 4 shows the results of these stability tests. Here, the extracted samples stored for 241 days show high stability for PDA-66. On the other hand, a profound loss was observed at the low concentration of PDA-66 when the extracted samples were stored at 23 °C or 6 °C for 24 h. In contrast, the plasma samples were also stable over the investigated storage period of 241 days at −80 °C, but showed a loss after 24 h of storage at 23 °C. At 6 °C, on the other hand, these samples were stable for 24 h. To avoid the losses due to the degradation of the substance at room temperature, all samples should nevertheless be processed quickly.

### 2.6. Quantification of PDA-66 in Mouse Plasma and Sera

To apply the method, 10 µL of artificial mouse plasma spiked with PDA-66 (5 µM) was mixed with human plasma to a final volume of 1 mL, resulting in a final concentration of 50 nM PDA-66. After the appropriate extraction of the sample, PDA-66 was successfully detected and quantified by LC-MS/MS. The experiment was performed three times. The mean of the three experiments gave a concentration of PDA-66 of 41.21 nM, with a CV of 10.50%.

As a further application of the validated method, mouse sera from two 129S4-Rag2^tm1.1Flv^Il2rg^tm1.1Flv^/J mice treated with PDA-66 (100 mg/kg body weight) intraperitoneally and euthanised after 3 h were analysed. Again, it was possible to quantify PDA-66. The corresponding analyses were carried out here in sera (200 µL each) from mice. The determined concentrations of PDA-66 were 6.93 µM and 8.25 µM, respectively. Due to the low plasma volume available, only single measurements per sample were performed.

## 3. Materials and Methods

### 3.1. Chemical Reagents

PDA-66 was used as 10 mM stock solution in DMSO with a purity of ≥98%. Acridine orange and LiChrosolv^®^ water were obtained from Merck Millipore (Darmstadt, Germany), formic acid from Honeywell Fluka (Seelze, Germany), S9 liver fraction (mouse and human) and lyophilised mouse plasma from Merck Millipore (Darmstadt, Germany) and acetonitrile ultragradient HPLC grade from J. T. Baker (Gliwice, Poland).

### 3.2. Standard Preparation

Calibration curves of PDA-66 ranging from 2.5 nM to 100 nM (2.5, 5, 7.5, 10, 15, 25, 50, 75 and 100 nM) were obtained by analysing 1 mL of human plasma mixed with 10 μL of an appropriate working solution. These were prepared from the PDA-66 stock solution (10 mM, dissolved in DMSO) by serial dilution in acetonitrile. Subsequently, 10 µL of a 5 µM working solution of the internal standard acridine orange (dissolved in water, final concentration 50 nM) was pipetted into the samples. The standard solutions were extracted three times with 1 mL extraction solution. The first step was performed using only ethyl acetate as the extraction solution, while the other two steps were performed with a mixture of ethyl acetate/n-hexane (2:1, *v*/*v*). Before and between the extractions, the samples were stored on ice to prevent temperature-dependent degradation. The organic phase was evaporated to dryness using a vacuum concentrator (SpeedVac SPD130DLX, Thermo Fisher Scientific, Asheville, NC, USA) and the residues were reconstituted in 100 μL water/acetonitrile (65:35, *v*/*v*) containing 0.2% formic acid for further analysis. To ensure the complete resolution of PDA-66 over the entire calibration range and of the internal standard, the evaporated samples were reconstituted with 100 µL of the solvent mixture.

### 3.3. LC-MS/MS Analysis

In total, 80 µL of the extracted samples were analysed on an Alliance Separations Module 2695 (Waters, Milford, MA, USA) HPLC using a Multospher 120 RP C18 AQ column 125 × 2 mm, 5 µm particle size coupled with a guard column 20 mm × 3 mm, 5 µm particle size (both from CS-Chromatographie Service GmbH, Langerwehe, Germany). The PDA-66 and the internal standard were separated with water containing 0.2% formic acid (mobile phase A) and acetonitrile containing 0.2% formic acid (mobile phase B) at a flow rate of 0.3 mL/min and a column temperature of 40 °C. The following procedure was chosen for the elution of the substances: the starting gradient was held at 65% mobile phase A for 2 min, then mobile phase B was linearly increased to 100% over 20 min, after which the starting conditions were immediately adjusted and held for a further 13 min for re-equilibration. Due to the gradient, metabolites of PDA-66 can be detected in subsequent experiments without changing the method. The HPLC system used was connected to a Micromass Quattro Micro^TM^ API mass spectrometer (Waters, Milford, MA, USA). The eluted substances were ionised by ESI in positive mode. Multiple reaction monitoring (MRM) was used to identify and quantify the analytes. The MS/MS transitions for the internal standard acridine orange at *m/z* 266.11 with a cone voltage of 24 V were *m/z* 234.1 with 44 V collision energy and *m/z* 250.1 with 24 V collision energy. For PDA-66, the following transitions were used: *m/z* 359.03 with cone voltage 26 V, and the fragments *m/z* 230.2 with 38 V collision energy and *m/z* 317.1 with 18 V collision energy. The quantifier for PDA-66 was *m/z* 317.1 and for the internal standard *m/z* 250.1. Both ions were the most abundant signals after fragmentation. The other two ions were used as qualifiers. Other mass spectrometer values and source parameters are listed in Table 3.

Argon was used as the collision gas for collision-induced dissociation (CID). The instrument parameters for the internal standard as well as for PDA-66 were set by injecting a 10 µM standard solution at a flow rate of 10 µL min^−1^ using a syringe pump. Data acquisition was performed using MassLynx version 4.1 software (Waters, Milford, MA, USA) and LabSolutions version 5.57 (Shimadzu, Duisburg, Germany).

### 3.4. Validation

Method validation was performed according to the FDA guidelines for bioanalytical methods of May 2018 [6]. The analytical parameters of calibration curve, selectivity, specificity, sensitivity, accuracy, precision, recovery, matrix effect and stability were addressed. Human plasma was chosen as the matrix because PDA-66 and the internal standard acridine orange are exogenous substances. The parameters were validated by evaluating the ratios of the daughter ion signals of the analyte peak and the internal standard peak. The calibration values were compared using the least squares method.

Calibration curves were generated using concentrations of PDA-66 ranging from 2.5 nM to 100 nM (2.5 nM, 5 nM, 7.5 nM, 10 nM, 15 nM, 25 nM, 50 nM, 75 nM, 100 nM). In accordance with FDA guidelines, 75% of these concentrations and at least 6 non-zero calibrators were used for the calibration curves. Depending on the parameter, PDA-66 was used in the concentrations of 7.5 nM (low concentration), 50 nM (medium concentration) or 100 nM (high concentration). The concentration of the internal standard acridine orange was adjusted to 50 nM in each run. The peak area ratios of the MS/MS fragments of the internal standard and the PDA-66 quantifiers were determined and linear regression was performed.

Six different human plasmas were analysed for selectivity. In order to meet the specificity required by the FDA guideline [6], the possible presence of interfering substances that could signal in addition to PDA-66 had to be excluded. The focus was placed on possible metabolites of PDA-66. For this purpose, a 10% murine and human S9 liver lysate solution was prepared according to the manufacturer’s instructions. PDA-66 was added to obtain a final concentration of 10 µM, and the sample was incubated for 3 h at 37 °C with gentle shaking. A sample spiked with dimethyl sulfoxide (DMSO) was included as a reference. The two samples were then extracted as described above and prepared for LC-MS analysis. To exclude the possibility of potential metabolites affecting the retention time of PDA-66 and the internal standard, a full scan in the range of *m/z* 250–750 was chosen.

The sensitivity was determined by evaluating the LLOQ with an S/N of at least 5 [6] using the software MassLynx version 4.1 without smoothing.

For accuracy, represented as relative error (RE; [(measured concentration—nominal concentration)/nominal concentration] × 100%), a deviation of ± 20% for LLOQ and ± 15% of its nominal concentrations for other concentrations used was allowed [6]. As a precision measure, the CV (standard deviation/mean of the measured concentration × 100%) was set at ± 20% for the LLOQ and ± 15% for other concentrations used, in accordance with FDA guidelines [6]. Five samples were determined for intra-day accuracy and precision. In comparison, for inter-day accuracy and precision, five samples each were measured on three consecutive days.

The matrix effect was determined by analysing five samples each of low (7.5 nM), medium (50 nM) and high (100 nM) concentrations. For this purpose, the matrix was extracted and spiked with PDA-66 or the internal standard. These samples were compared with non-extracted standards of the two compounds as presented here: [1 − (postspiked signal/stock solution signal)] × 100%. The absolute values of the areas were compared.

To determine the recovery of PDA-66, at least five independent plasma samples were used. Recovery was assessed for samples spiked with low (7.5 nM), medium (50 nM) and high (100 nM) concentrations prior to extraction. The extracted matrix samples spiked with the three concentration levels after extraction were analysed as reference values. Recovery was determined by comparing the peak areas of the pre-spiked extracted samples and the peak areas of the post-spiked extracted matrix samples, according to the following calculation: (sample signal/postspiked signal) × 100%. The internal standard was added prior to analysis. For the recovery of the internal standard, the absolute values of the areas were also compared.

Stability was determined as short-term and long-term stability. Long-term stability was determined by storing plasma spiked with analytes at −80 °C for 241 days and extracted samples at −80 °C for 90 days. Short-term stability was analysed for both extracted and evaporated as well as non-extracted plasma samples by storage for 24 h at 23 °C and 6 °C. The peak area curves of the analyte and the internal standard of the stability experiments were compared with those of samples freshly prepared on the day of measurement. The internal standard was added to the extracted and evaporated as well as the non-extracted stored samples and their reference samples immediately before analysis. To all other samples, the internal standard was added before sample processing. However, to avoid concentration fluctuations due to the possible degradation of PDA-66, stock solutions were prepared once a month and stored at −20 °C.

### 3.5. Preparation of Mouse Plasma and Sera

Since PDA-66 is not yet approved for use in humans, the method could not be evaluated in treated patients. Instead, artificial mouse plasma and sera from treated mice were used.

In the latter case, sera of two 129S4-Rag2^tm1.1Flv^Il2rg^tm1.1Flv^/J mice treated with PDA-66 were examined. The mice were purchased from the Jackson Laboratory (Bar Harbour, ME), and bred in the local animal facility (Rostock University Medical Centre) under specified pathogen-free conditions. The health status of the animals was routinely checked according to FELASA guidelines. All mice were exposed to a 12 h artificial light–dark cycle and kept in cages under ad libitum access to water and standard laboratory chow. Both mice were treated intraperitoneally with 100 mg/kg PDA-66 and euthanised after 3 h. Blood was then collected in a 1.5 mL Eppendorf tube and sera were obtained by centrifugation (10 min, 2000× *g*).

In the case of the measurement of PDA-66 in artificial mouse plasma, the lyophilisate of the plasma was resuspended in LiChrosolv^®^ water and PDA-66 was added to obtain a final concentration of 5 µM.

For PDA-66 analysis, 10 µL of artificial mouse plasma supplemented with PDA-66 or 200 µL of serum from each of the PDA-66-treated mice was added to human plasma with a resulting final volume of 1 mL. The identical human plasma was used for dilution of the artificial mouse plasma and mouse sera, and for the calibration function. This procedure was chosen to minimise the matrix effect of the human plasma. The mixture was then processed, extracted and analysed with the internal standard as already described. The PDA-66 concentrations measured in the sera of the treated mice, which were diluted with human plasma before extraction, were finally converted into 1 mL of pure mouse serum.

## 4. Conclusions

Targeted antitumour therapy with small molecules has been complementing conventional therapy for several years. PDA-66 is a new substance in this field with anticancer potential. Monitoring the concentration of such substances in human plasma is one of the key factors when assessing pharmacokinetics as well as therapeutic success. The method is characterised by a low LLOQ of 2.5 nM, which shows that little material will be needed for determination in plasma samples in the future. The recovery of PDA-66 from human plasma was about 80 to 100%, depending on the concentration, meaning a large amount of the substance can be recovered by simple liquid–liquid extraction. Therefore, a validated method for the sensitive determination of the concentration of PDA-66 was presented. The method was successfully used for the quantification of PDA-66 in mouse plasma and sera.

## Figures and Tables

**Table 1:** Inter-day and intra-day accuracy, expressed as relative error (RE) ± standard deviation (SD), and precision, determined as the coefficient of variation (CV), of five different samples each from three days of PDA-66 at LLOQ (2.5 nM), low (7.5 nM), medium (50 nM) and high (100 nM) concentrations. The measured concentrations are given as mean values ± SD, *n* = 5 for intra-day, *n* = 15 for inter-day.

Used Concentration (nM)	Intra-Day (Day 1)			Intra-Day (Day 2)			Intra-Day (Day 3)			Inter-Day	
	Measured Concentration (nM)	RE (%)	CV (%)	Measured Concentration (nM)	RE (%)	CV (%)	Measured Concentration (nM)	RE (%)	CV (%)	RE (%)	CV (%)
2.5	2.30 ± 0.2	−8.04 ± 9.1	9.89	2.56 ± 0.2	2.33 ± 7.9	7.73	2.61 ± 0.2	4.45 ± 8.8	8.44	−0.42 ± 9.8	9.82
7.5	6.78 ± 0.4	−9.62 ± 4.7	5.20	7.26 ± 0.6	−3.15 ± 8.3	8.54	7.68 ± 0.7	2.34 ± 8.9	8.66	−3.48 ± 8.6	8.91
50	48.46 ± 4.5	−3.08 ± 9.0	9.23	46.81 ± 2.3	−6.38 ± 4.5	4.82	47.83 ± 2.4	−4.35 ± 4.7	4.91	−4.60 ± 6.1	6.37
100	96.64 ± 10.0	−3.36 ± 10.0	10.32	109.62 ± 5.3	9.62 ± 5.3	4.79	102.68 ± 7.6	2.68 ± 7.6	7.37	2.98 ± 9.1	8.83

**Table 2:** Matrix effect and recovery of PDA-66 and the internal standard acridine orange. Values are mean percentages ± standard deviation (SD) of *n =* 5 (except mid and high recovery levels, *n* = 10).

PDA-66			Acridine Orange		
Concentration (nM)	Recovery (%)	Matrix Effect (%)	Concentration (nM)	Recovery (%)	Matrix Effect (%)
7.5	102.0 ± 6.8	31.0 ± 9.1	50	65.89 ± 5.3	21.4 ± 5.8
50	79.0 ± 7.4	28.0 ± 9.3			
100	85.0 ± 6.7	28.3 ± 14.7			

**Table 3:** Applied mass spectrometer parameters and source parameters.

Parameters for MS Instrument	
Capillary voltage	3.5 kV
Source temperature	120 °C
Desolvation temperature	350 °C
Desolvation gas flow rate	550 L h^−1^
Cone gas flow rate	50 L h^−1^
Dwell times	0.1 s
Delay times	0.1 s

## Data Availability

The data presented in this study are available on reasonable request from the first author.

## Sample Availability

Not available.
